# Supplementary material for: A split and inducible adenine base editor for precise in vivo base editing
Source: Nat Commun. 2023 Sep 11;14:5573. doi: 10.1038/s41467-023-41331-5 (PMC10495389; doi:10.1038/s41467-023-41331-5)
Supplement: Supplementary file 1 — Supplementary Information [file 41467_2023_41331_MOESM1_ESM.pdf]

# Supplementary Information

## A split and inducible adenine base editor for precise in vivo base editing

Hongzhi Zeng[1], Qichen Yuan[1†], Fei Peng[2†], Dacheng Ma[1†], Ananya Lingineni[3], Kelly Chee[4], Peretz Gilberd[4], Emmanuel C. Osikpa[1], Zheng Sun[2,5*], and Xue Gao[1,3,6*]

[1]Department of Chemical and Biomolecular Engineering, Rice University, Houston, Texas 77005, USA

[2]Department of Medicine, Division of Diabetes, Endocrinology and Metabolism, Baylor College of Medicine, Houston, Texas 77030, USA

[3]Department of Bioengineering, Rice University, Houston, Texas 77005, USA

[4]Department of Biosciences, Rice University, Houston, Texas 77005, USA

[5]Department of Molecular and Cellular Biology, Baylor College of Medicine, Houston, Texas 77030, USA

[6]Department of Chemistry, Rice University, Houston, TX 77005, USA

†These authors contributed equally.

* To whom correspondence should be addressed: xue.gao@rice.edu (X.G.), zheng.sun@bcm.edu (Z.S.)

# Table of Contents

## *Supplementary Figures*

**Supplementary Fig. 1 |** Flow cytometry gating strategies for the EYFP reporter assay.

**Supplementary Fig. 2 |** Engineered sABEs with optimized split site and CID domains.

**Supplementary Fig. 3 |** Engineered sABEs with the optimized split site, CID domains, and linkers.

**Supplementary Fig. 4 |** Rapamycin-dependent control of sABE v3.22 activity.

**Supplementary Fig. 5 |** Genomic DNA on-target editing efficiencies, activity window and product purity of sABE v3.22 and ABE8e.

**Supplementary Fig. 6 |** Adaption of sABE v3.22 architecture to TadA8e variants F148A and V106W.

**Supplementary Fig. 7 |** Adaption of sABE v3.22 architecture to SaCas9 nickase and dead Un1Cas12f1 (CasMINIv3.1, CasMINIV4).

**Supplementary Fig. 8 |** Cas9-dependent and Cas9-independent off-target effects of ABEs.

**Supplementary Fig. 9 |** Transcriptomic off-target effects of ABEs.

**Supplementary Fig. 10 |** Flow cytometry gating strategies for sorting the top 5% GFP positive cells.

**Supplementary Fig. 11 |** Flow cytometry gating strategies for HEK293T cells stained with anti-B2M antibodies.

**Supplementary Fig. 12 |** Flow cytometry gating strategies for HEK293T cells stained with anti-CD46 antibodies.

**Supplementary Fig. 13 |** Protein expression of the target genes in HEK293T cells.

**Supplementary Fig. 14 |** Triple AAVs and triple lentivirus delivery of sABE v3.22.

**Supplementary Fig. 15 |** In vitro and in vivo editing at m*PCSK9* intron 1 splice donor site.

# *Supplementary Figures*

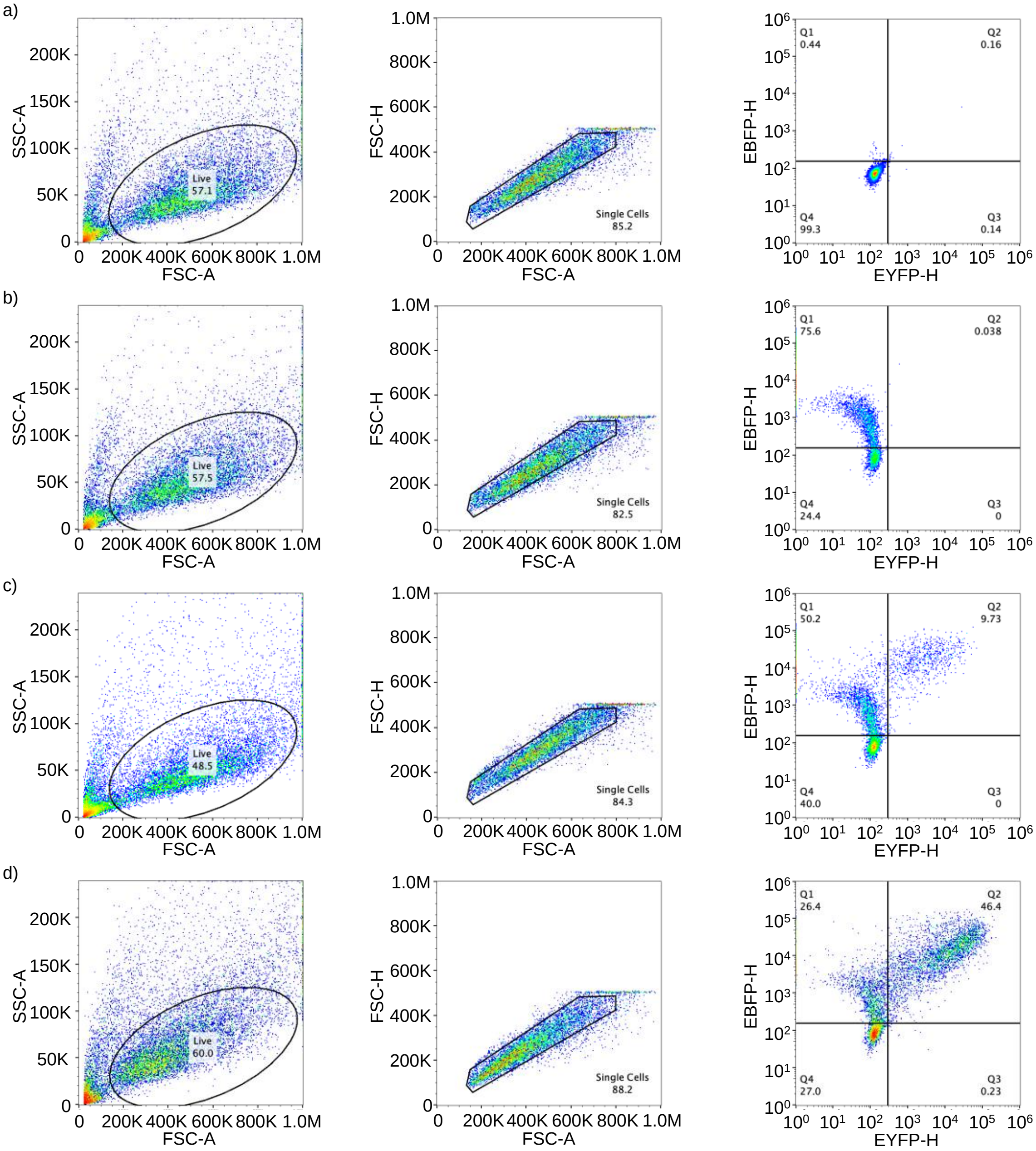


**Supplementary Fig. 1 | Flow cytometry gating strategies for the EYFP reporter assay.** Live cells were gated by side scatter area versus forward scatter area (SSC-A vs. FSC-A). Singlets were selected by forward scatter height versus forward scatter area (FSC-H vs. FSC-A). Fluorescence-positive population was gated against the mock-transfected control with EBFP height versus EYFP height (EBFP-H vs. EYFP-H). **a)** HEK293T cells transfected with plasmids encoding EYFP* and sgRNA. **b)** with plasmids encoding EYFP*, sgRNA, and EBFP. **c)** with plasmids encoding EYFP*, sgRNA, EBFP, and sABE v2; without rapamycin induction. **d)** with plasmids encoding EYFP*, sgRNA, EBFP, and sABE v2; with 100nM rapamycin induction.

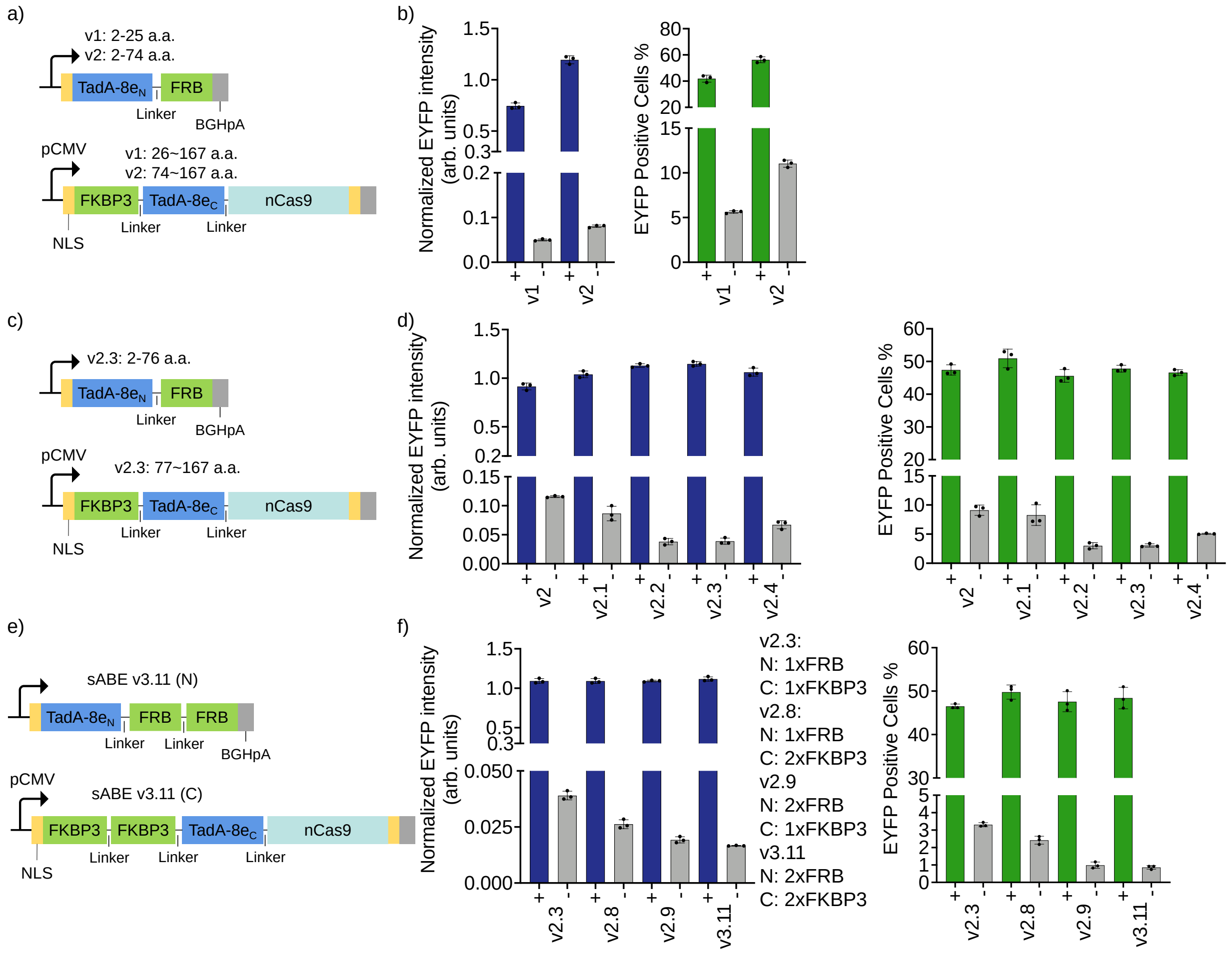


**Supplementary Fig. 2 | Engineered sABEs with optimized split site and CID domains. a)** Diagram of sABE v1 or v2 constructs. **b)** EYFP* reporter responses to sABE v1 or v2. Left: Normalized EYFP intensity. Right: Percentage of EYFP-positive cells. Blue or green: with 100 nM rapamycin induction; grey: non-induced. **c)** Diagram of sABE v2.3 constructs. **d)** EYFP* reporter responses to sABE v2 (split after arginine 74), v2.1 (split after tryptophan 73), v2.2 (split after leucine 75), v2.3 (split after isoleucine 76), and v2.4 (split after asparagine 77). Left: Normalized EYFP intensity. Right: Percentage of EYFP-positive cells. **e)** Diagram of sABE v3.11 constructs. **f)** EYFP* reporter responses to sABE v2.3, v2.8, v2.9, or v3.11. Left: Normalized EYFP intensity. Right: Percentage of EYFP-positive cells. In **b)**, **d)**, and **f)**, dots represent data from three independent biological replicates, and bars represent their mean with s.d.

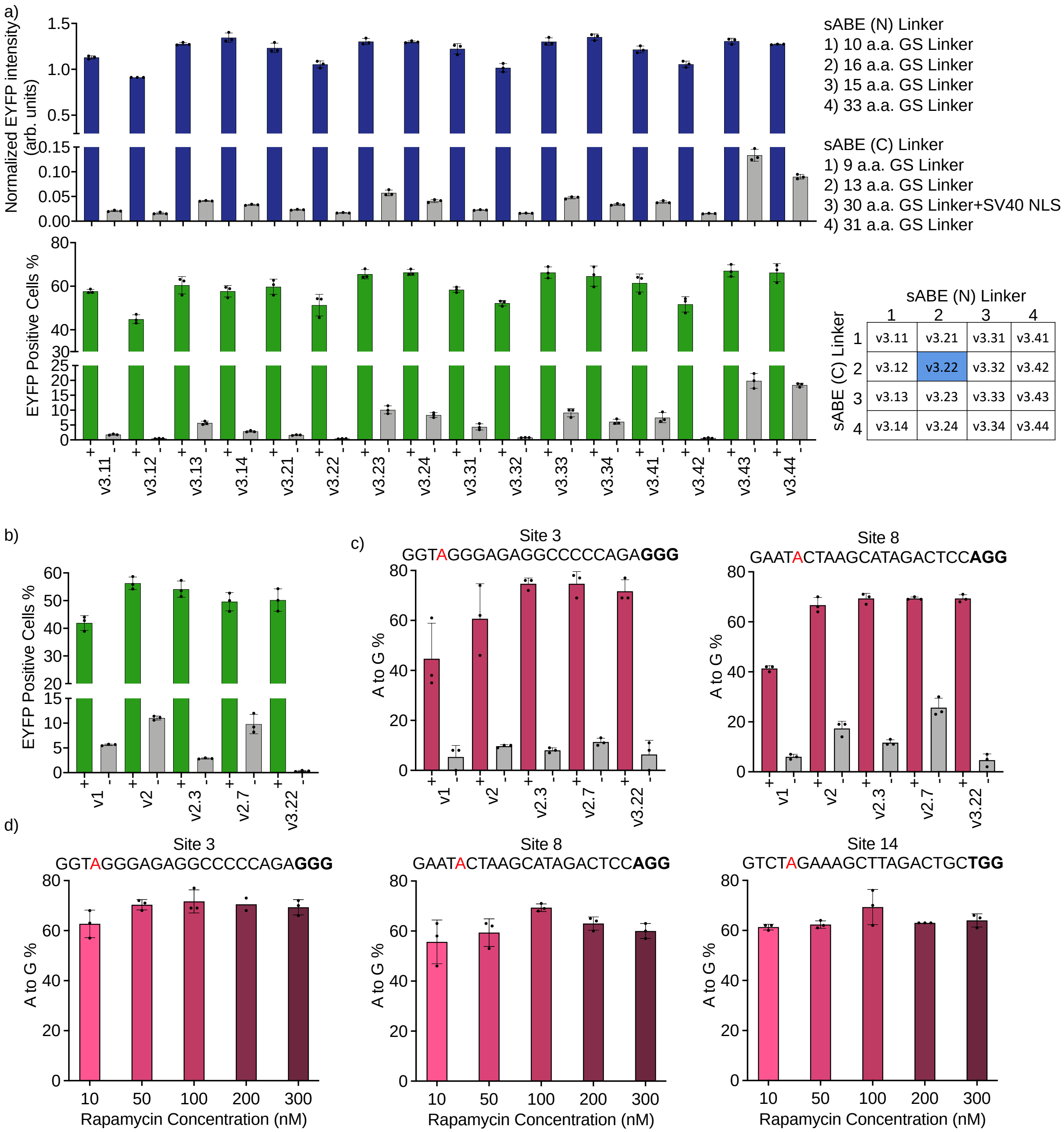


**Supplementary Fig. 3 | Engineered sABEs with the optimized split site, CID domains, and linkers.** **a)** EYFP* reporter responses to sABE v3.11-v3.44. Top left: Normalized EYFP intensity. Bottom left: Percentage of EYFP-positive cells. Blue or green: with 100 nM rapamycin induction; grey: non-induced. Top right: Engineered linkers for sABE(N) and sABE(C). Bottom right: Engineered Linkers used in sABE v3.11 -3.44. sABE v3.22 is highlighted in blue. **b)** Percentage of EYFP-positive cells from the EYFP* reporter assay by 5 selected versions of sABEs. **c)** A-to-G base editing efficiencies of the highlighted adenine by five versions of sABEs at genomic Site 3 and Site 8. Red: with 100 nM rapamycin induction; grey: non-induced. **d)** A-to-G base editing efficiencies of the highlighted adenine at Site 3, 8, and 14 by sABE v3.22 with rapamycin induction ranging from 10 nM to 300 nM. Editing efficiencies in **c)** and **d)** are evaluated by Sanger sequencing. In all subpanels, dots represent three individual biological replicates (except for the 200 nM group at Site 3 in **d** where n=2), and bars represent mean ± s.d.

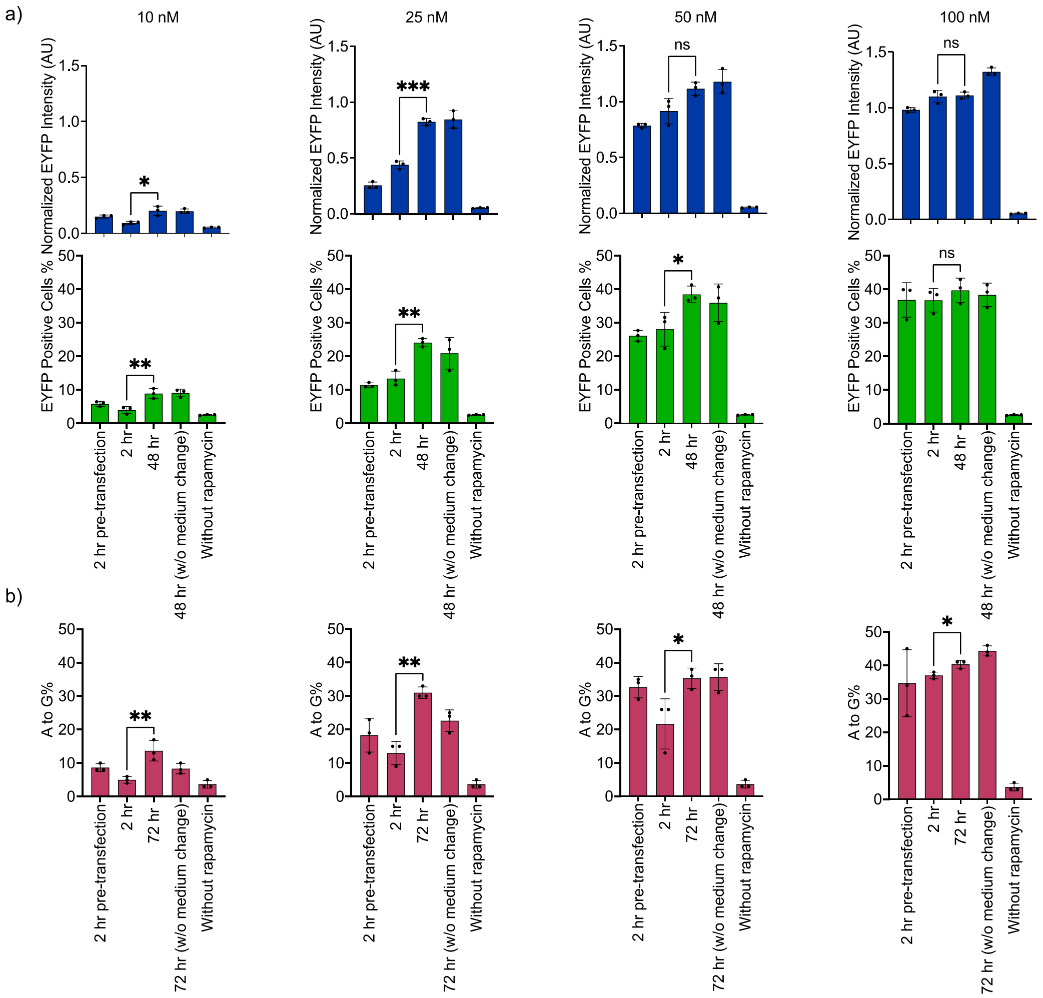


**Supplementary Fig. 4 | Rapamycin-dependent control of sABE v3.22 activity. a)** EYFP* reporter responses to sABE v3.22 under different rapamycin concentration and exposure time. Top: Normalized EYFP intensity. The P-values from left to right are 0.0128, 0.0001, 0.0533, 0.8033. Bottom: Percentage of EYFP-positive cells. The P-values are from left to right are 0.0097, 0.0019, 0.0326, 0.3728. **b)** A-to-G conversion rates evaluated by Sanger sequencing by sABE v3.22 at Site 14 under different rapamycin concentration and exposure time. The P-values from left to right are 0.0095, 0.0013, 0.0432, 0.0194. In all subpanels, dots represent three individual biological replicates, and bars represent mean ± s.d. All subpanels use unpaired two-tailed t-test; ns, not significant; *P < 0.05; **P < 0.01; ***P < 0.001; ****P < 0.0001).

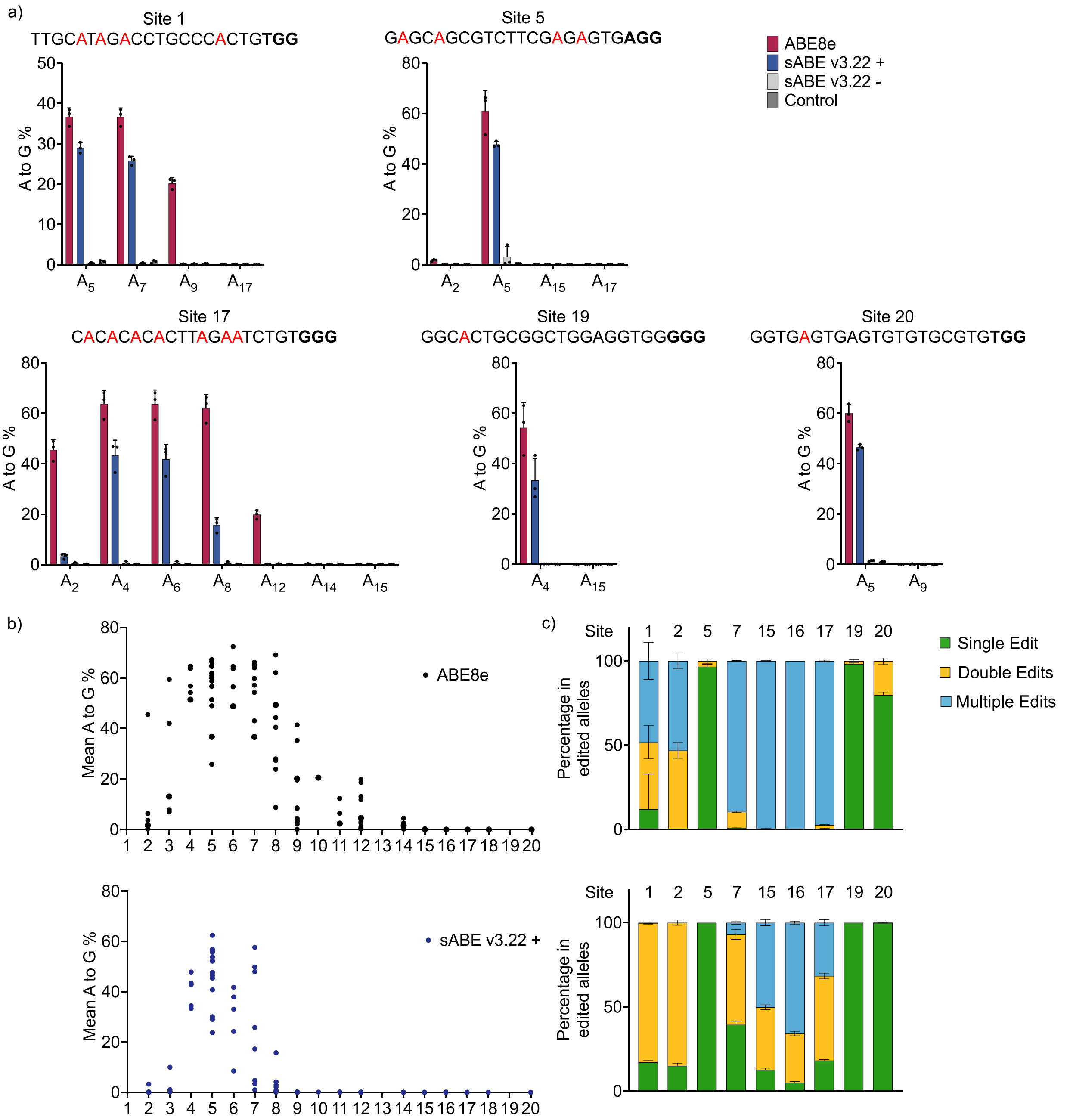


**Supplementary Fig. 5 | Genomic DNA on-target editing efficiencies, activity window and product purity of sABE v3.22 and ABE8e. a)** Bar charts show the on-target A-to-G base editing efficiencies of ABE8e, sABE v3.22 induced with 100nM rapamycin, and uninduced sABE v3.22 at Site 1, 5, 17, 19, and 20. Control group was mock-transfected. Dots represent three individual biological replicates, and bars represent mean ± s.d. **b)** Mean A-to-G base editing efficiencies of ABE8e (Top) and sABE v3.22 (Bottom) at 19 tested loci. Each dot represents the mean value of A-to-G base editing efficiencies on adenine at each genomic site displayed in **a)** and Figure 2a. Dots are divided into bins based on the position of their corresponding adenine in the protospacer. **c)** Bar plot showing the percentage of reads containing different numbers of base conversions in ABE8e edited alleles (Top) or in sABE v3.22 edited alleles (Bottom) at genomic Site 1, 2, 5, 7, 15, 16, 17, 19, and 20. Colors represent if the reads contain single (green), double (yellow), or multiple (blue) A-to-G conversions. Bars represent mean ± s.d. of three individual biological replicates.

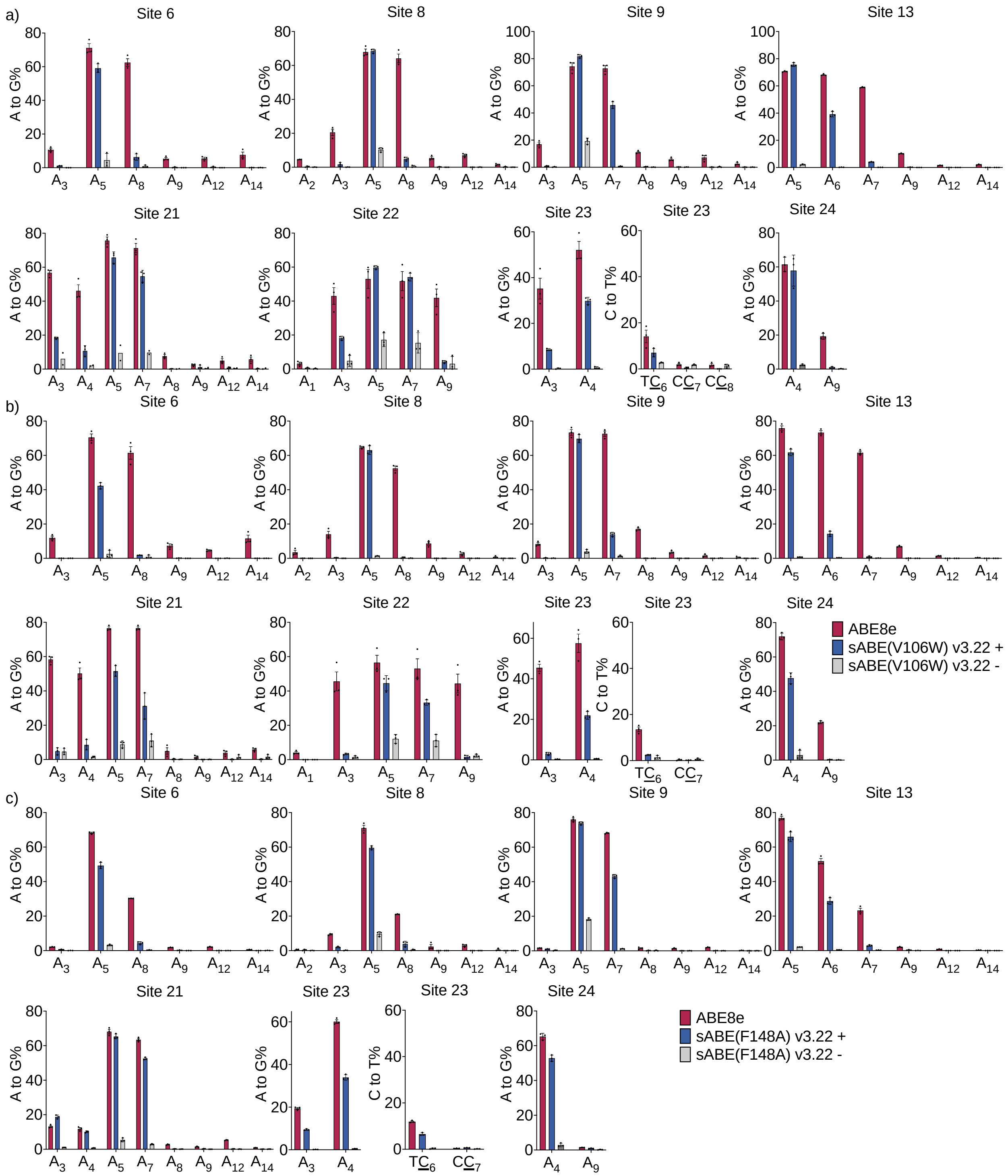


**Supplementary Fig. 6 | Adaption of sABE v3.22 architecture to TadA8e variants F148A and V106W.** **a)** A-to-G base editing efficiencies of ABE8e, sABE v3.22 induced with 100nM rapamycin, and uninduced sABE v3.22 at eight genomic loci. **b)** A-to-G base editing efficiencies of ABE8e(V106W), sABE(V106W) v3.22 induced with 100nM rapamycin, and uninduced sABE(V106W) v3.22 at eight genomic loci. **c)** A-to-G base editing efficiencies of ABE8e(F148A), sABE(F148A) v3.22 induced with 100nM rapamycin, and uninduced sABE(F148A) v3.22 at seven genomic loci. In all subpanels, dots represent individual biological replicates (except for **a)** Site 21 sABE v3.22 – group where n=2), and bars represent mean ± s.d.

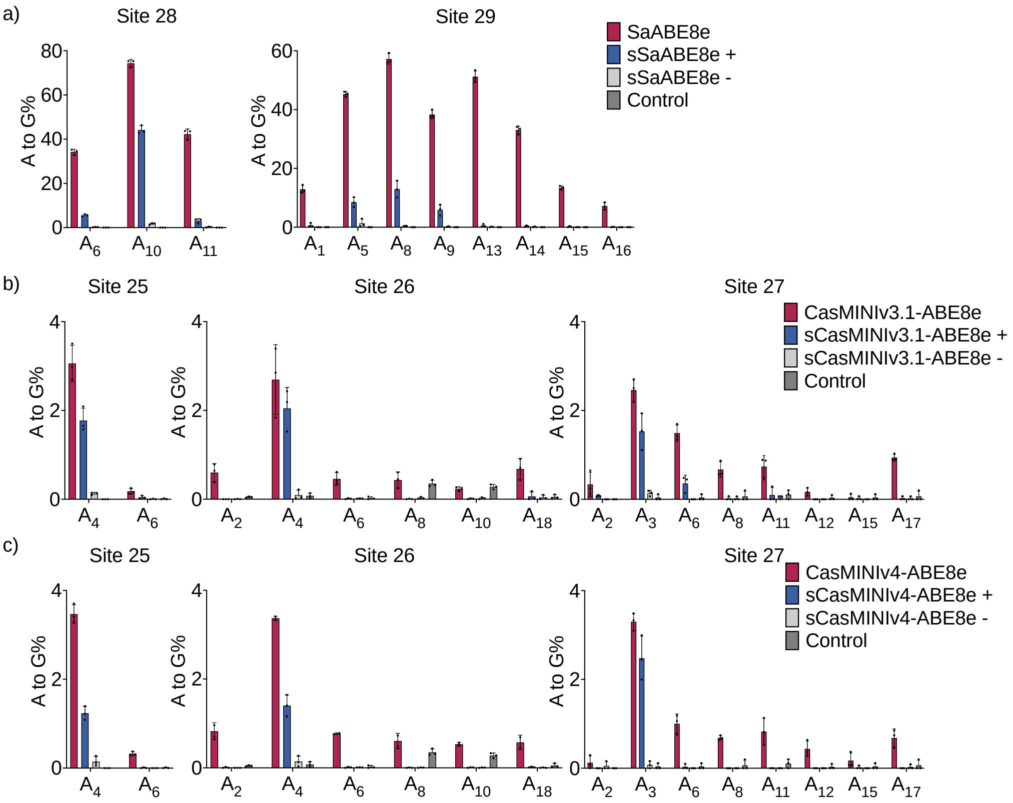


**Supplementary Fig. 7 | Adaption of sABE v3.22 architecture to SaCas9 nickase and dead Un1Cas12f1 (CasMINIv3.1, CasMINIV4). a)** A-to-G base editing efficiencies of SaABE8e, sSaABE8e induced with 100nM rapamycin, and uninduced sSaABE8e at two genomic loci. **b)** A-to-G base editing efficiencies of CasMINIv3.1-ABE8e, sCasMINIv3.1-ABE8e induced with 100nM rapamycin, and uninduced sCasMINIv3.1-ABE8e at three genomic loci. **c)** A-to-G base editing efficiencies of CasMINIv4-ABE8e, sCasMINIv4-ABE8e induced with 100nM rapamycin, and uninduced sCasMINIv4-ABE8e at three genomic loci. In all subpanels, dots represent three individual biological replicates, and bars represent mean ± s.d.

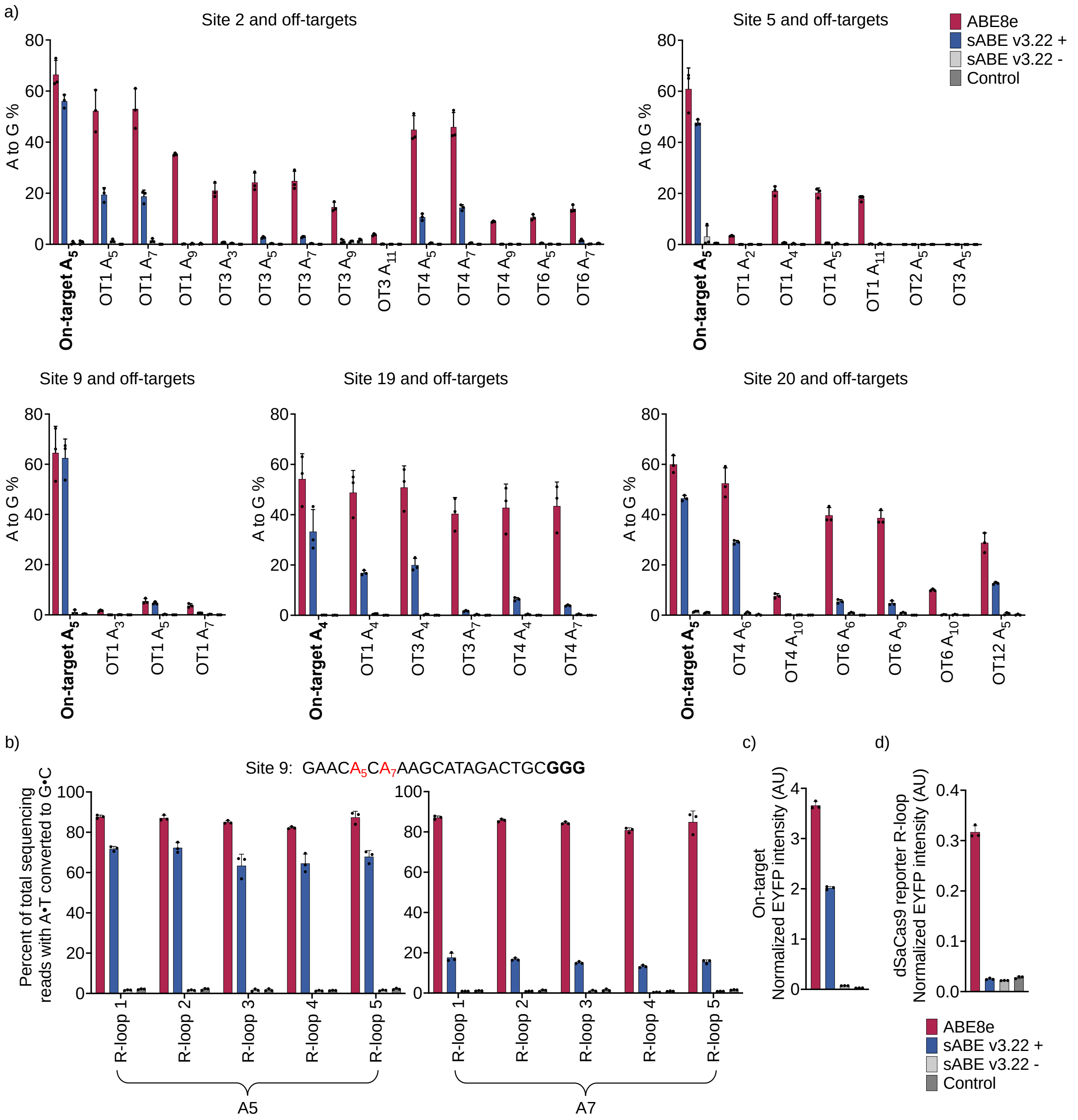


**Supplementary Fig. 8 | Cas9-dependent and Cas9-independent off-target effects of ABEs. a)** A-to-G conversions at five DNA on-target loci and fourteen of their Cas9-dependent DNA off-target loci in HEK293T cells transfected with ABE8e or sABE v3.22. sABE v3.22 was induced with 100 nM rapamycin. **b)** DNA on-target editing efficiencies by ABE8e or sABE v3.22 in HEK293T cells at site 9 in the orthogonal R-loop assay. sABE v3.22 was induced with 100 nM rapamycin. HTS reads consisting of <0.2% of total reads were not considered. **c)**, **d)** The EYFP reporter assay repurposed as a reporter orthogonal R-loop assay. HEK293T cells were transfected with either **c)** EYFP* reporter, ABE, and a SpCas9 sgRNA activating the EYFP* reporter or with **d)** EYFP* reporter, base editor, dSaCas9, and a SaCas9 sgRNA targeting the same site. sABE v3.22 was induced with 100 nM rapamycin. In all subpanels, dots represent three individual biological replicates, and bars represent mean ± s.d. HTS reads consisting of <0.2% of total reads were not considered. OT: off-target.

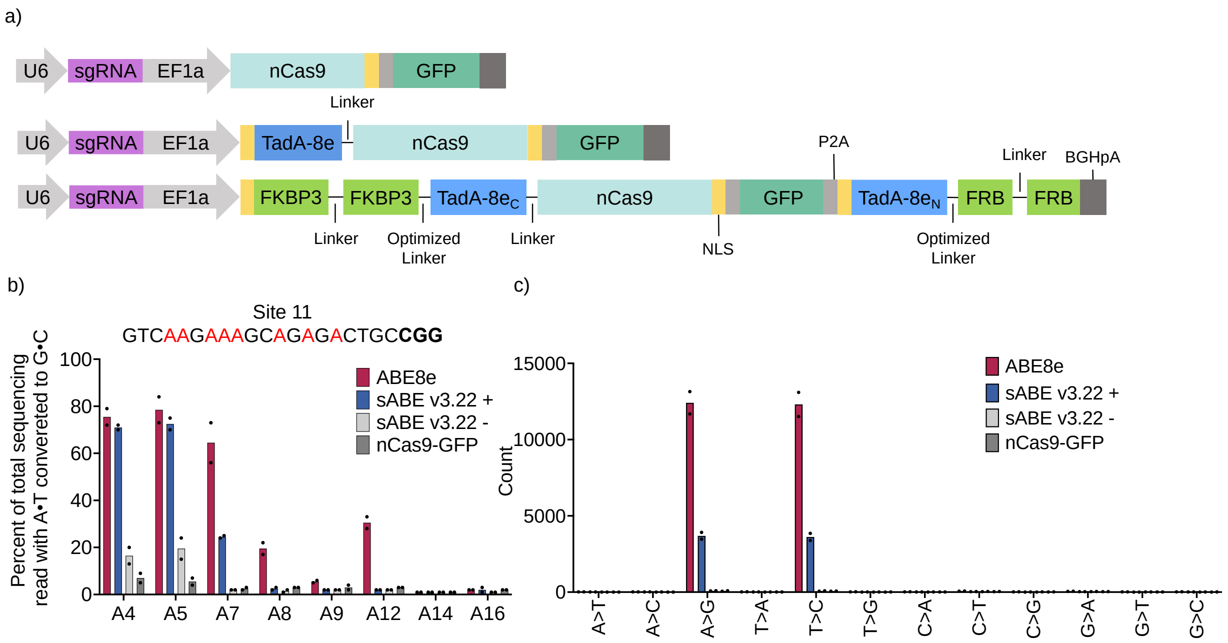


**Supplementary Fig. 9 | Transcriptomic off-target effects of ABEs. a)** Plasmid design for the RNA-seq experiment. Top: nCas9-P2A-GFP; Middle: ABE8e-P2A-GFP; Bottom: All-In-One sABE v3.22. Each plasmid also contains a sgRNA targeting the genomic site 11. **b)** DNA on-target A-to-G base editing efficiencies by ABE8e or sABE v3.22 or nCas9 in transfected HEK293T cells with top 5% GFP fluorescence. sABE v3.22 was induced with 100 nM rapamycin. Editing efficiencies were validated via Sanger sequencing. All adenines in the protospacer are highlighted red and the PAM is emboldened. **c)** Quantitative analysis of all 12 types of SNVs in the final filtered VCF. In **b)** and **c)**, dots represent two individual biological replicates and bars represent mean.

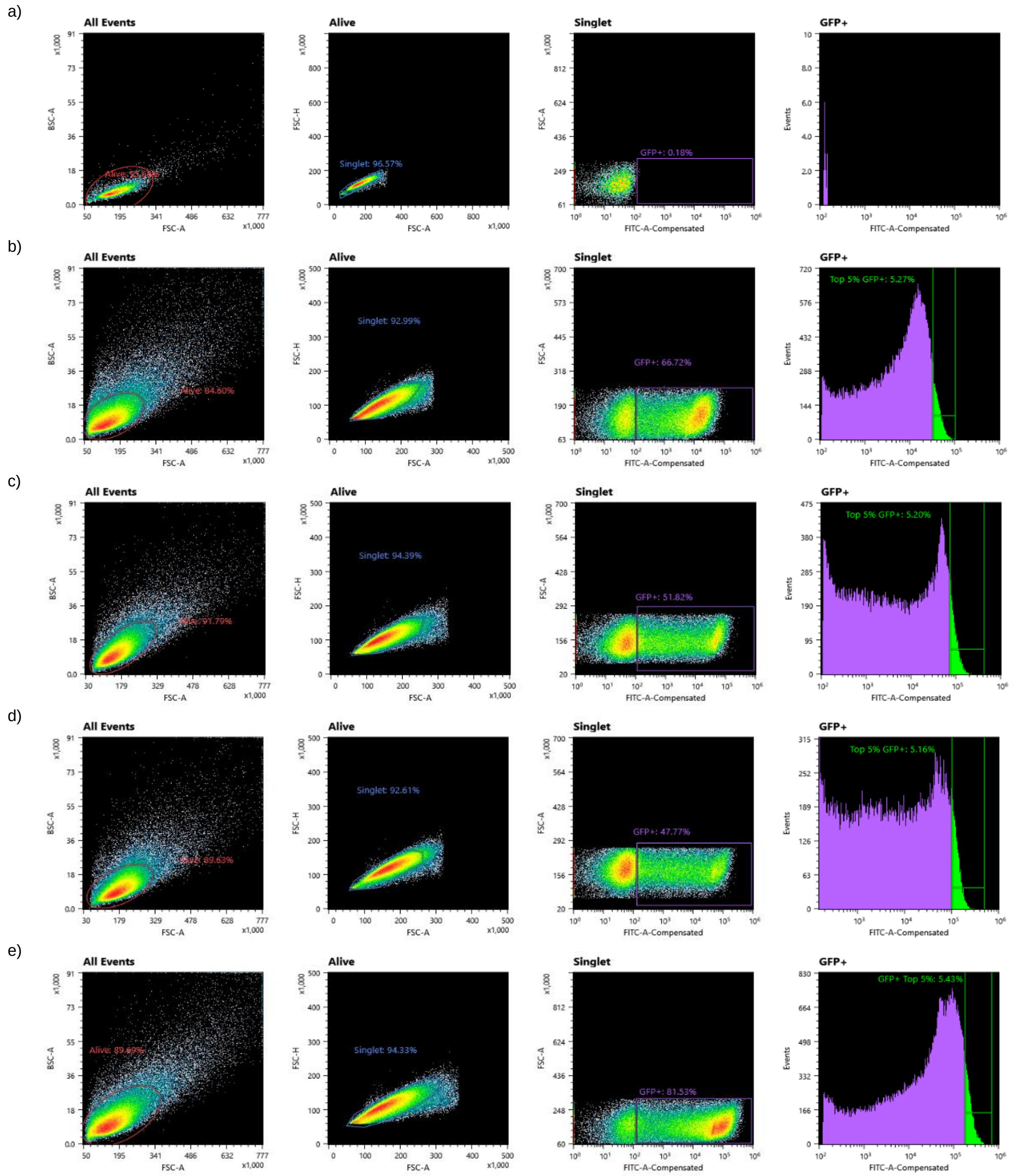


**Supplementary Fig. 10 | Flow cytometry gating strategies for sorting the top 5% GFP positive cells.** Live cells were gated by back scatter area versus forward scatter area (BSC-A vs. FSC-A). Singlets were selected by forward scatter height versus forward scatter area (FSC-H vs. FSC-A). Fluorescence-positive population was gated against the mock-transfected control. **a)** Mock-transfected HEK293T cells. **b)** HEK293T cells transfected with ABE8e-P2A-GFP targeting Site 11. **c)** HEK293T cells transfected with All-In-One sABE v3.22 targeting Site 11 without rapamycin induction. **d)** HEK293T cells transfected with All-In-One sABE v3.22 targeting Site 11 with 100 nM rapamycin induction. **e)** HEK293T cells transfected with nCas9-P2A-GFP targeting Site 11.

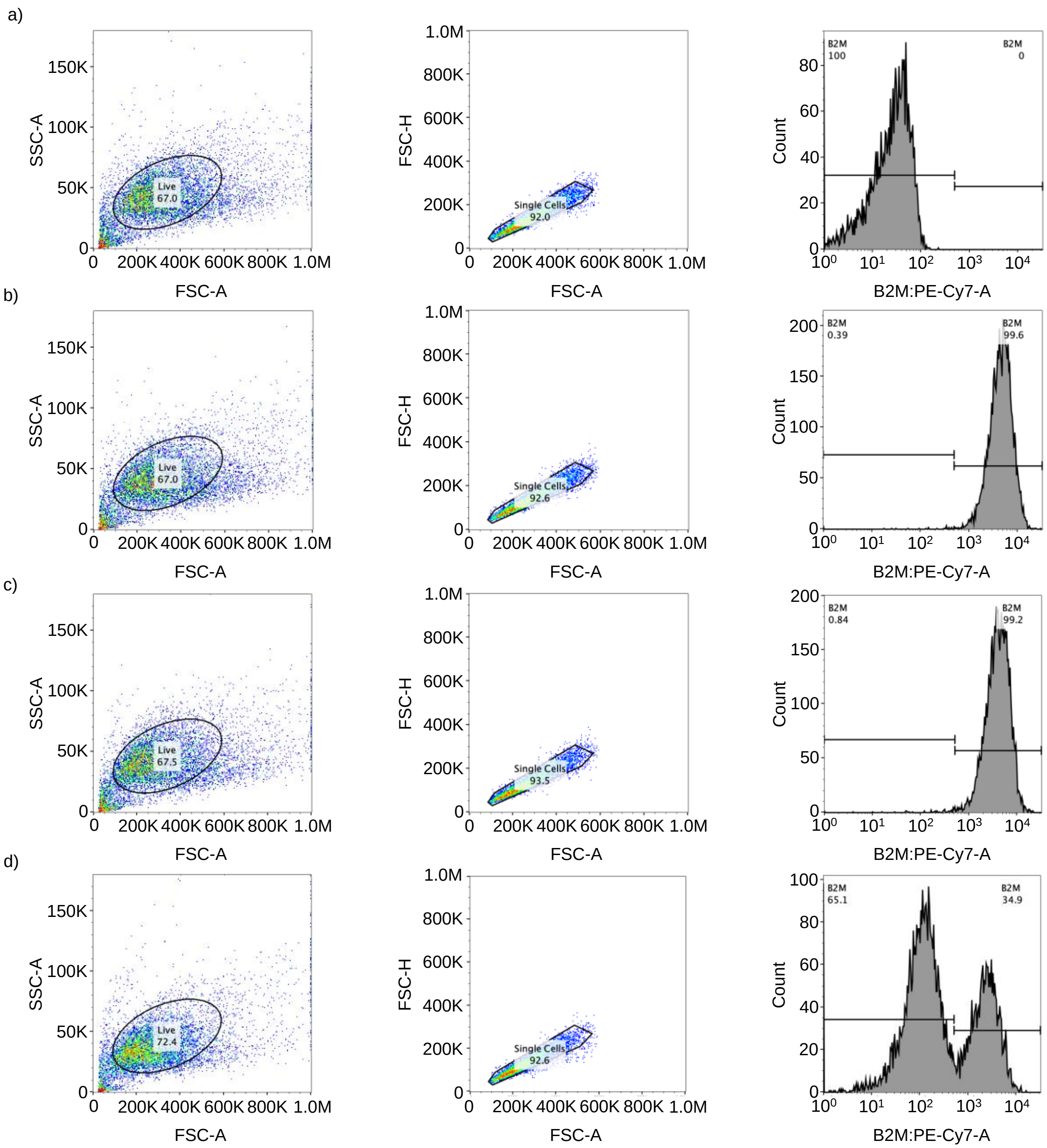


**Supplementary Fig. 11 | Flow cytometry gating strategies for HEK293T cells stained with anti-B2M antibodies.** Live cells were selected by side scatter area versus forward scatter area (SSC-A vs. FSC-A). Singlets were gated by forward scatter height versus forward scatter area (FSC-H vs. FSC-A). Fluorescence-positive population was gated against the mock-transfected control. **a)** Mock-transfected and unstained HEK293T cells. **b)** Mock-transfected HEK293T cells, stained by PE/Cyanine7 anti-human B2M antibodies. **c)** HEK293T cells transfected with sABEv3.22 and DAP-MBE array targeting B2M splice donors without rapamycin induction, stained by PE/Cyanine7 anti-human B2M antibodies. **d)** HEK293T cells transfected with sABEv3.22 and DAP-MBE array targeting B2M splice donors with 100 nM rapamycin induction, stained by PE/Cyanine7 anti-human B2M antibodies.

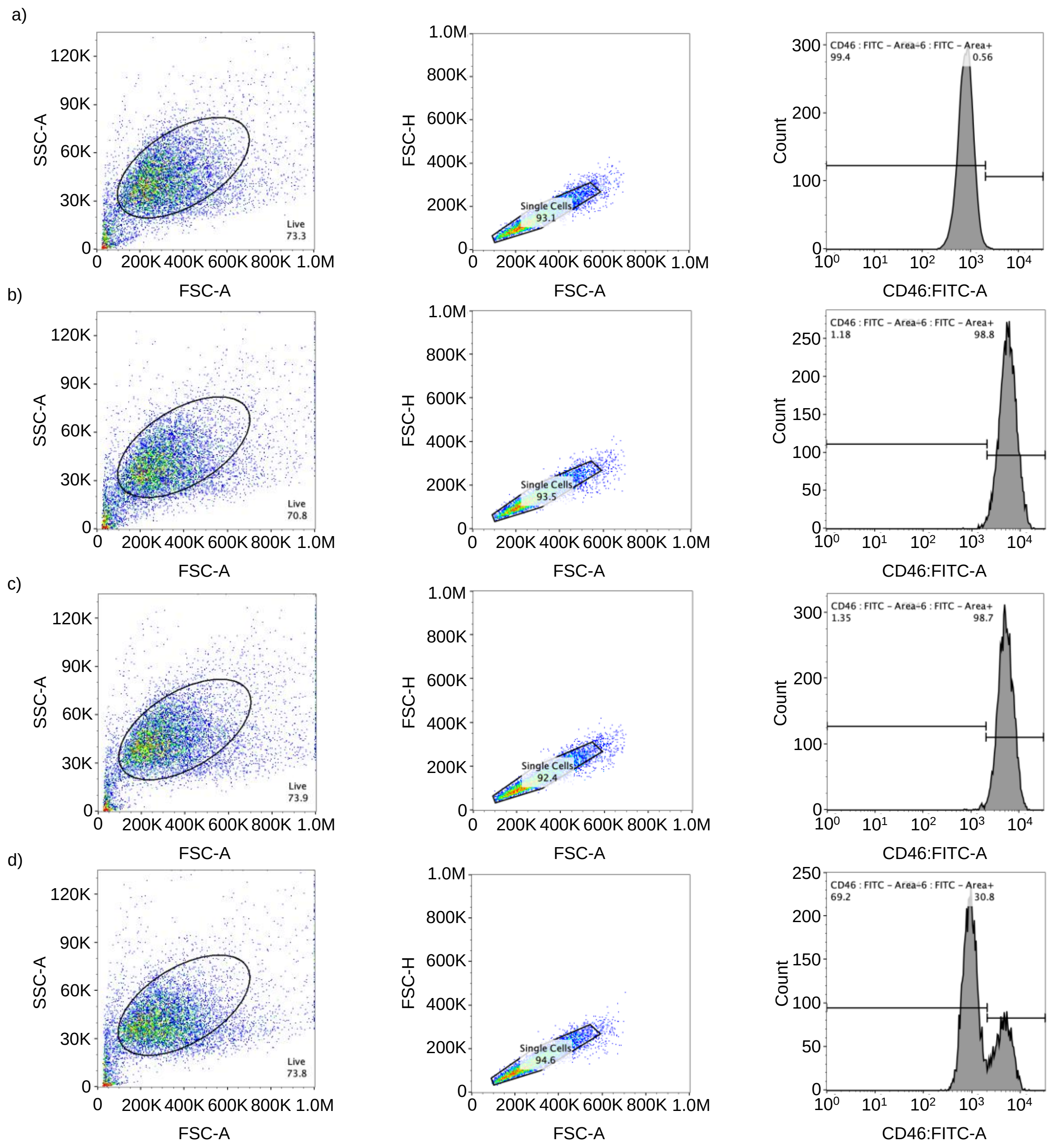


**Supplementary Fig. 12 | Flow cytometry gating strategies for HEK293T cells stained with anti-CD46 antibodies** Live cells were selected by side scatter area versus forward scatter area (SSC-A vs. FSC-A). Singlets were gated by forward scatter height versus forward scatter area (FSC-H vs. FSC-A). Fluorescence-positive population was gated against the mock-transfected control. **a)** Mock-transfected and unstained HEK293T cells. **b)** Mock-transfected HEK293T cells, stained by FITC anti-human CD46 antibodies. **c)** HEK293T cells transfected with sABEv3.22 and DAP-MBE array targeting CD46 splice donors without rapamycin induction, stained by FITC anti-human CD46 antibodies. **d)** HEK293T cells transfected with sABEv3.22 and DAP-MBE array targeting CD46 splice donors with 100 nM rapamycin induction, stained by FITC anti-human CD46 antibodies.

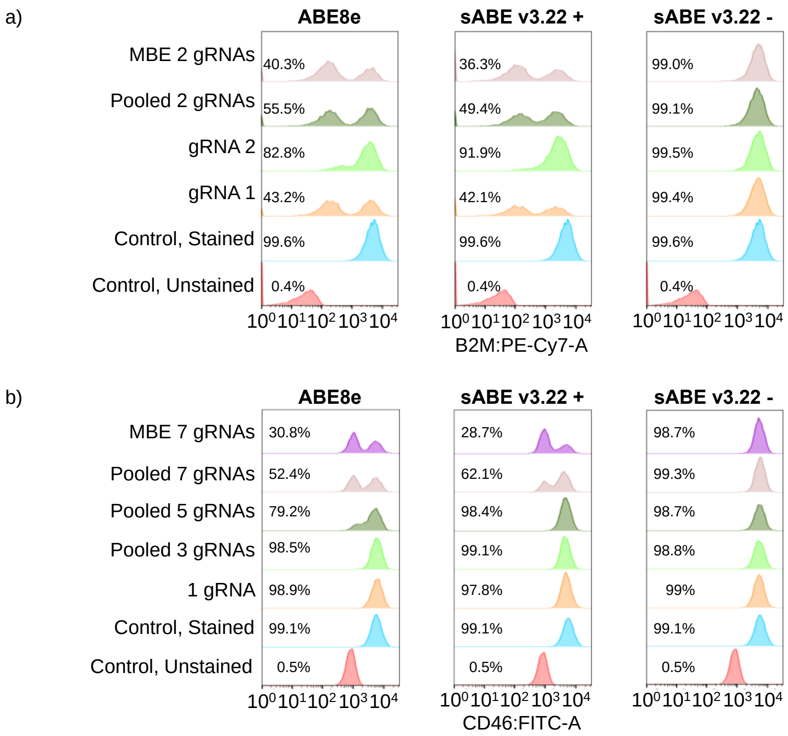


**Supplementary Fig. 13 | Protein expression of the target genes in HEK293T cells.** Protein expression from **a)** B2M gene and **b)** CD46 gene in HEK293T cell transfected with sgRNAs or DAP-MBE array targeting splice donors and sABE v3.22 or ABE8e. Percentage of B2M- or CD46- positive cells was quantified using antibody-based FACS analysis. Representative flow cytometry data shown here came from one replicate from each group. Complete sets of biological replicates are shown in Fig. 4. Controls were mock-transfected.

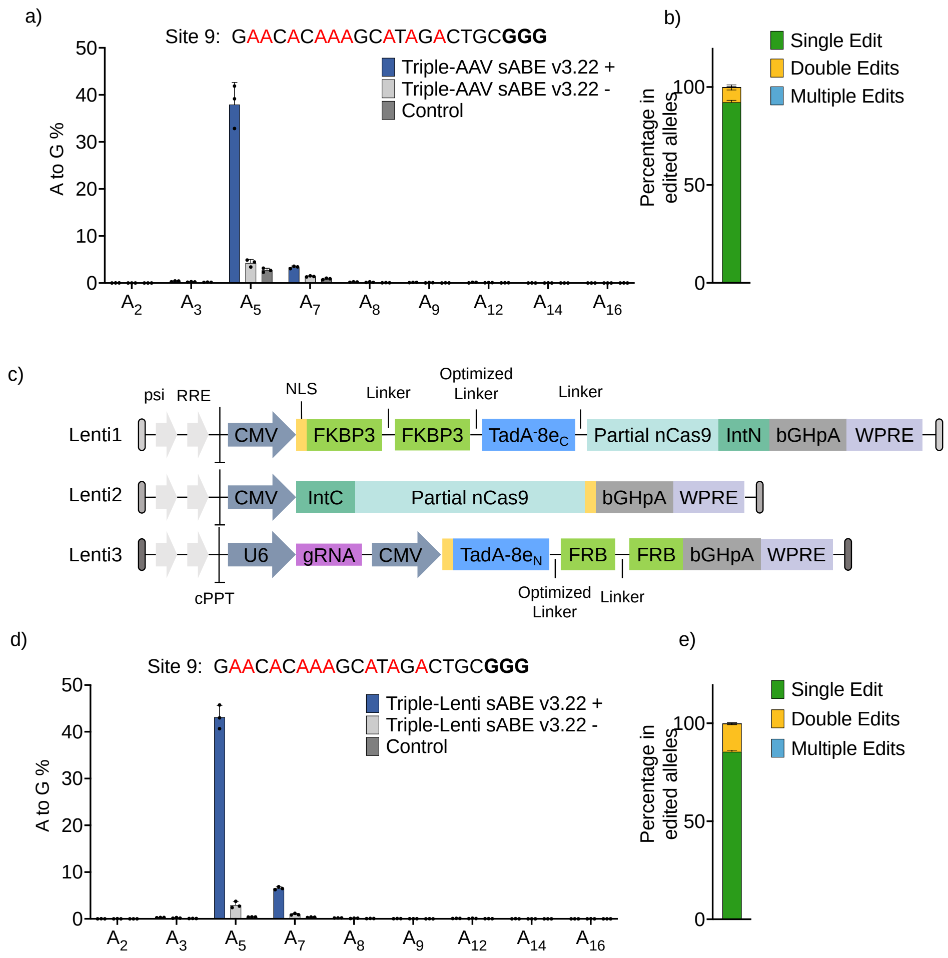


**Supplementary Fig. 14 | Triple AAVs and triple lentivirus delivery of sABE v3.22. a)** A-to-G base editing efficiencies at Site 9 in HEK293T cells transduced with triple AAVs encoding sABE v3.22 and a sgRNA targeting Site 9. **b)** Product purity of all edited alleles in the triple-AAVs transduced group with 100 nM rapamycin induction. **c)** Triple lentiviruses encoding sABE v3.22. bGHpA: bovine growth hormone polyadenylation signal. WPRE: Woodchuck hepatitis virus posttranscriptional regulatory element. **d)** A-to-G base editing efficiencies at Site 9 in HEK293T cells transduced with triple lentiviruses encoding sABE v3.22 and a sgRNA targeting Site 9. **e)** Product purity of all edited alleles in the triple-lentiviruses transduced group with 100 nM rapamycin induction. In **a)** and **d)**, dots represent three individual biological replicates. In **a)**, **b)**, **d)**, and **e)**, bars represent mean ± s.d of three individual biological replicates. HTS reads consisting of <0.2% of total reads were not considered. All adenines in the protospacer are highlighted red, and the PAM is emboldened.

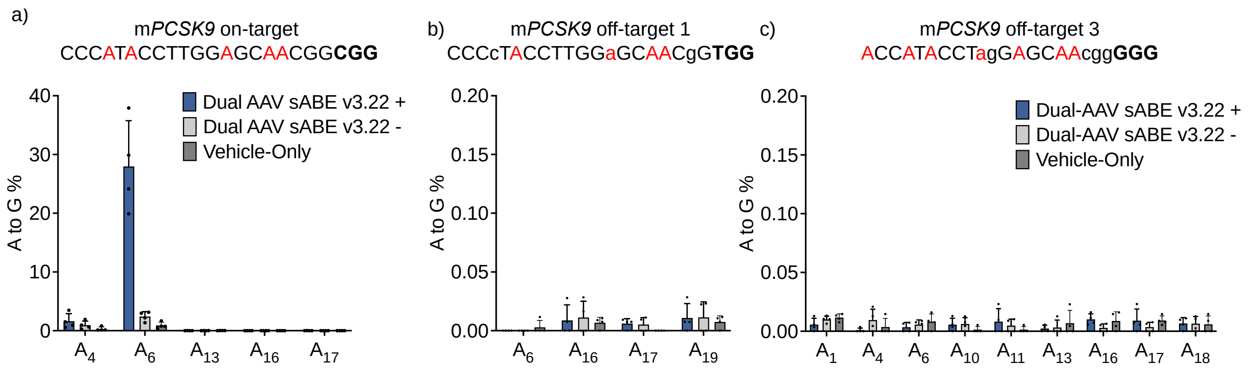


**Supplementary Fig. 15 | In vitro and in vivo editing at m*PCSK9* intron 1 splice donor site. a)** A-to-G base editing efficiencies at m*PCSK9* intron 1 splice donor site in mice liver. DNA was extracted from mouse liver tissue. n=4 animals in each group. All adenines in the protospacer are highlighted in red, and the PAM is emboldened. Vehicle-Only: Mock-injected with vehicle. **b)**, **c)** A-to-G conversions on the Cas9-dependent off-targets of m*PCSK9* intron 1 splice donor site. **b)** off-target 1 or **c)** off-target 3. Lowercased letters in the sequences above represent mismatches of the off-target sites with the on-target site. In all subpanels, dots represent four individual biological replicates, and bars represent mean ± s.d. HTS reads consisting of <0.2% of total reads were not considered.
