# Supplementary material for: A split and inducible adenine base editor for precise in vivo base editing
Source: Nat Commun. 2023 Sep 11;14:5573. doi: 10.1038/s41467-023-41331-5 (PMC10495389; doi:10.1038/s41467-023-41331-5)
Supplement: Supplementary file 2 — Reporting Summary [file 41467_2023_41331_MOESM2_ESM.pdf]

nature portfolio

Corresponding author(s): Xue Gao, Zheng Sun

Last updated by author(s): Aug 11, 2023

Reporting Summary

Nature Portfolio wishes to improve the reproducibility of the work that we publish. This form provides structure for consistency and transparency in reporting. For further information on Nature Portfolio policies, see our Editorial Policies and the Editorial Policy Checklist.

Statistics

For all statistical analyses, confirm that the following items are present in the figure legend, table legend, main text, or Methods section.

n/a	Confirmed	
☐	☒	The exact sample size (*n*) for each experimental group/condition, given as a discrete number and unit of measurement
☐	☒	A statement on whether measurements were taken from distinct samples or whether the same sample was measured repeatedly
☐	☒	The statistical test(s) used AND whether they are one- or two-sided<br>*Only common tests should be described solely by name; describe more complex techniques in the Methods section.*
☒	☐	A description of all covariates tested
☐	☒	A description of any assumptions or corrections, such as tests of normality and adjustment for multiple comparisons
☐	☒	A full description of the statistical parameters including central tendency (e.g. means) or other basic estimates (e.g. regression coefficient) AND variation (e.g. standard deviation) or associated estimates of uncertainty (e.g. confidence intervals)
☐	☒	For null hypothesis testing, the test statistic (e.g. *F*, *t*, *r*) with confidence intervals, effect sizes, degrees of freedom and *P* value noted<br>*Give P values as exact values whenever suitable.*
☒	☐	For Bayesian analysis, information on the choice of priors and Markov chain Monte Carlo settings
☒	☐	For hierarchical and complex designs, identification of the appropriate level for tests and full reporting of outcomes
☒	☐	Estimates of effect sizes (e.g. Cohen's *d*, Pearson's *r*), indicating how they were calculated

Our web collection on statistics for biologists contains articles on many of the points above.

Software and code

Policy information about availability of computer code

Data collection	Illumina Miseq; Illumina NextSeq 550; Sony Biotechnology SA3800 Spectral Cell Analyzer; Sony Biotechnology MA900 multi-application cell-sorter
Data analysis	Flowjo (version 10.8.1); GraphPad Prism (version 9.4.1); CRISPResso2 (version 2.2.9, https://github.com/pinellolab/CRISPResso2); EditR (v1.0.0, https://github.com/MoriarityLab/EditR); GATK Best Practices for RNA-seq short variant discovery tools, including MarkDuplicates (version 2.27.4), SplitNCigarReads (version 4.2.6.1), BaseRecalibrator (version 4.2.6.1), HaplotyperCaller(version 4.2.6.1), and selectVariant (version 4.2.6.1) (https://github.com/broadinstitute/gatk); STAR (version 2.7.10a, https://github.com/alexdobin/STAR) ; bam-readcount (version 1.0.1, https://github.com/genome/bam-readcount); PyMOL (version 2.5.2).

For manuscripts utilizing custom algorithms or software that are central to the research but not yet described in published literature, software must be made available to editors and reviewers. We strongly encourage code deposition in a community repository (e.g. GitHub). See the Nature Portfolio guidelines for submitting code & software for further information.

Data

Policy information about availability of data

All manuscripts must include a data availability statement. This statement should provide the following information, where applicable:

- Accession codes, unique identifiers, or web links for publicly available datasets
- A description of any restrictions on data availability
- For clinical datasets or third party data, please ensure that the statement adheres to our policy

High-throughput DNA- and RNA-Seq data are deposited at the Sequence Read Archive (PRJNA923001): https://www.ncbi.nlm.nih.gov/bioproject/PRJNA923001. Source data of each figure and nucleic acid sequences of all constructs used in this study are available in the Supplementary Tables. Structure of TadA-8e can be found in Protein Data Bank (PDB: 6VPC, https://www.rcsb.org/structure/6vpc).

Human research participants

Policy information about studies involving human research participants and Sex and Gender in Research.

Reporting on sex and gender	N/A
Population characteristics	N/A
Recruitment	N/A
Ethics oversight	N/A

Note that full information on the approval of the study protocol must also be provided in the manuscript.

Field-specific reporting

Please select the one below that is the best fit for your research. If you are not sure, read the appropriate sections before making your selection.

☒ Life sciences ☐ Behavioural & social sciences ☐ Ecological, evolutionary & environmental sciences

For a reference copy of the document with all sections, see nature.com/documents/nr-reporting-summary-flat.pdf

Life sciences study design

All studies must disclose on these points even when the disclosure is negative.

Sample size	Sample sizes were determined by observed variability across independent experiments. No statistical method was used to predetermine sample size. No data were excluded from the analyses. These sizes align with common practices in related research.
Data exclusions	No data were excluded from the study.
Replication	All measurements reported in the study were collected in at least two independent biological replicates with cells from different passages or batches. All independent biological replicates were successful and produced consistent results. No data were excluded from the analyses.
Randomization	Randomization was not employed in the cell line assay due to the use of consistent batches of cell lines and maintained uniform culture conditions throughout the study. We rigorously controlled for variables such as cell density, passage number, media composition, and incubation times. By controlling all major experimental variables, observed effects can be attributed to the experimental treatments and not variations within the cell lines themselves. For animal experiments, at 15 weeks of age, the twelve C57BL/6 male mice were randomly divided into three groups, with four mice in each group. The mice then underwent specific experimental treatments based on their group assignments.
Blinding	The investigators were not blinded to allocation during experiments and outcome assessment. The data were processed and analyzed in exactly the same way. There were no subjective decisions or interpretations made during the data analysis phase.

Reporting for specific materials, systems and methods

We require information from authors about some types of materials, experimental systems and methods used in many studies. Here, indicate whether each material, system or method listed is relevant to your study. If you are not sure if a list item applies to your research, read the appropriate section before selecting a response.

Materials & experimental systems

n/a	Involved in the study	
	x	Antibodies
	x	Eukaryotic cell lines
x		Palaeontology and archaeology
	x	Animals and other organisms
x		Clinical data
x		Dual use research of concern

Methods

n/a	Involved in the study	
x		ChIP-seq
	x	Flow cytometry
x		MRI-based neuroimaging

Antibodies

Antibodies used: FITC anti-human CD46 Antibody (BioLegend, Catalog 315304, Lot B339203, Clone MEM-258); PE/Cy7 anti-human β2-microglobulin Antibody (BioLegend, Catalog 316318, Lot B371988, Clone 2M2)

Validation: Both antibodies were validated by the suppliers by staining using isotype controls. Specific details are listed below for each antibody.
FITC anti-human CD46 Antibody (BioLegend #315304): https://www.biolegend.com/en-us/products/fitc-anti-human-cd46-antibody-2782?GroupID=BLG4970
E/Cy7 anti-human β2-microglobulin Antibody (BioLegend #316318): https://www.biolegend.com/en-us/products/pe-cyanine7-anti-human-beta2-microglobulin-antibody-13839?GroupID=BLG4462

Eukaryotic cell lines

Policy information about cell lines and Sex and Gender in Research

Cell line source(s): HEK293T: American Type Cell Culture Collection (ATCC) CRL-3216

Authentication: Authenticated by the supplier using STR analysis.

Mycoplasma contamination: All cells used were tested negative for mycoplasma contamination using Myco-Blue Mycoplasma Detector (Vazyme).

Commonly misidentified lines (See ICLAC register): The cell line used is not listed as commonly misidentified.

Animals and other research organisms

Policy information about studies involving animals; ARRIVE guidelines recommended for reporting animal research, and Sex and Gender in Research

Laboratory animals: Twelve male C57BL/6 mice, aged 15 weeks, were utilized for the experiments, sourced from the Jackson Laboratory.

Wild animals: No wild animal was used in the study.

Reporting on sex: A total of 12 C57BL/6 mice were involved in the animal experiment.

Field-collected samples: No field-collected sample was used in the study.

Ethics oversight: All mice were maintained and handled following laboratory animal treatments approved by the Institutional Animal Care and Use Committee (IACUC) of Baylor College of Medicine (BCM).

Note that full information on the approval of the study protocol must also be provided in the manuscript.

Flow Cytometry

Plots

Confirm that:

- [x] The axis labels state the marker and fluorochrome used (e.g. CD4-FITC).
- [x] The axis scales are clearly visible. Include numbers along axes only for bottom left plot of group (a 'group' is an analysis of identical markers).
- [x] All plots are contour plots with outliers or pseudocolor plots.
- [x] A numerical value for number of cells or percentage (with statistics) is provided.

Methodology

Sample preparation	1. EYFP reporter assay. 100 µl TrypLE Express (Thermo Fisher Scientific) was added to each well and was incubated at room temperature for 5 minutes to detach cells. 200 µl Dulbecco's Modified Eagle's Medium (DMEM) plus GlutaMAX (Gibco) supplemented with 10% (v/v) fetal bovine serum (Gibco) and 1% (v/v) penicillin-streptomycin (Gibco) was then added to each well, and the mixture was pipetted 30~40 times for cell suspension.<br><br>2. RNA-seq experiment. Cells transfected with EGFP-containing plasmid from each 10 cm plate were dissociated with 2 ml TrypLE Express, centrifuged at 400 × g for 3 minutes at room temperature, and resuspended in 5 ml complete media. 0.5 to 0.7E6 cells with the top 5 % GFP signal were sorted using the MA900 multi-application cell-sorter (Sony).<br><br>3. Inducible knockout experiments. Cells per 24-well were detached with 500µl TrypLE Express (Thermo Fisher Scientific) and were incubated at room temperature for 5 minutes. The suspended cells were pipetted firmly 5~10 times, transferred to 1.5 ml microcentrifuge tubes, and centrifuged at 500 × g for 3 minutes. The supernatant was discarded, and 500 µl cell staining buffer (BioLegend) was used to resuspend the cells. Cells were counted by Countess II FL (Thermo Fisher Scientific), and 2~3E5 cells were diluted in 100 µl cell staining buffer. 3 µl FITC anti-human CD46 antibody (BioLegend #315304) or PE/Cy7 anti-human β2-microglobulin antibody (BioLegend #316318) was mixed with the 100 µl cell suspension and was incubated in the dark on ice for 20 minutes. The supernatant was discarded, and the cells were washed with 500 µl cell staining buffer by centrifugation at 500 × g for 3 minutes. The final cell pellet was suspended in 500 µl cell staining buffer.
Instrument	SA3800 Spectral Cell Analyzer (Sony Biotechnology); MA900 multi-application cell-sorter (Sony)
Software	Flowjo 10.8.1
Cell population abundance	In RNA-sequencing experiment, 0.5 to 0.7E6 cells with the top 5 % GFP signal were sorted. This represent 2%-3% of total population due to FSC v.s. SSC and FSC-H v.s. FSC-A gating and the sorting yield of MA900.
Gating strategy	Cells were gated on their population via the forward versus side scatter (FSC vs. SSC) plot. The plot of forward scatter height versus forward scatter area (FSC-H vs. FSC-A) was used to exclude doublet. In 1. The double-gated population is plotted as EYFP-H v.s. EBFP-H plots and the quadrant gating is determined by the mock-transfected control and applied to all samples.<br>In 2. EYFP-positive population is gated on the mock-transfected control. The triple-gated population was plotted as histograms, and cells within top 5% EGFP-positive population were sorted.<br>In 3. The double-gated population was plotted as histograms.

☒ Tick this box to confirm that a figure exemplifying the gating strategy is provided in the Supplementary Information.
