# Supplementary material for: A split and inducible adenine base editor for precise in vivo base editing
Source: Nat Commun. 2023 Sep 11;14:5573. doi: 10.1038/s41467-023-41331-5 (PMC10495389; doi:10.1038/s41467-023-41331-5)
Supplement: Supplementary file 3 — Description of Additional Supplementary Files [file 41467_2023_41331_MOESM3_ESM.pdf]

**Title: Supplementary Data. 1**

**Description:** Constructs, descriptions, and maps.

**Title: Supplementary Data. 2**

**Description:** Information of tested genomic loci.

**Title: Supplementary Data. 3**

**Description:** Primers for high-throughput sequencing.

**Title: Supplementary Data. 4**

**Description:** Primers for Sanger sequencing.
